# COVID-19 instigates adipose browning and atrophy through VEGF in small mammals

**DOI:** 10.1038/s42255-022-00697-4

**Published:** 2022-12-08

**Authors:** Xu Jing, Jieyu Wu, Caijuan Dong, Juan Gao, Takahiro Seki, Changil Kim, Egon Urgard, Kayoko Hosaka, Yunlong Yang, Siwen Long, Ping Huang, Junnian Zheng, Laszlo Szekely, Yuanting Zhang, Wei Tao, Jonathan Coquet, Minghua Ge, Yuguo Chen, Mikael Adner, Yihai Cao

**Affiliations:** 1 Department of Microbiology, Tumor and Cell Biology, Karolinska Institute, Stockholm, Sweden; 2 Experimental Asthma and Allergy Research Unit, Institute of Environmental Medicine (IMM), Karolinska Institute, Stockholm, Sweden; 3 Department of Cardiovascular Medicine, First Affiliated Hospital of Xi’an Jiaotong University, Xi’an, China; 4 Department of Infectious Diseases, The Third Affiliated Hospital of Sun Yat-sen University, Guangzhou, China; 5 Department of Cellular and Genetic Medicine, School of Basic Medical Sciences, Fudan University, Shanghai, China; 6 Department of Pharmacy, Zhejiang Provincial People’s Hospital, People’s Hospital of Hangzhou Medical College, Hangzhou, China; 7 Key Laboratory of Endocrine Gland Diseases of Zhejiang Province, Hangzhou, China; 8 Cancer Institute, Xuzhou Medical University, Xuzhou, China; 9 Department of Pathology/Cytology, Karolinska University Laboratory, Stockholm, Sweden; 10 Department of Biomedical Engineering, City University of Hong Kong, Kowloon Tong, Hong Kong; 11 Hong Kong Centre for Cerebro-cardiovascular Health Engineering, Hong Kong, Hong Kong; 12 Center for Nanomedicine and Department of Anesthesiology, Brigham and Women’s Hospital, Harvard Medical School, Boston, MA USA; 13 Department of Head, Neck and Thyroid Surgery, Zhejiang Provincial People’s Hospital, People’s Hospital of Hangzhou Medical College, Hangzhou, China; 14 Department of Emergency Medicine, Shandong Provincial Clinical Research Center for Emergency and Critical Care Medicine, Institute of Emergency and Critical Care Medicine of Shandong University, Qilu Hospital of Shandong University, Jinan, China

**Keywords:** Fat metabolism, Infectious diseases, Angiogenesis

## Abstract

Patients with COVID-19 frequently manifest adipose atrophy, weight loss and cachexia, which significantly contribute to poor quality of life and mortality^1,2^. Browning of white adipose tissue and activation of brown adipose tissue are effective processes for energy expenditure^3–7^; however, mechanistic and functional links between SARS-CoV-2 infection and adipose thermogenesis have not been studied. In this study, we provide experimental evidence that SARS-CoV-2 infection augments adipose browning and non-shivering thermogenesis (NST), which contributes to adipose atrophy and body weight loss. In mouse and hamster models, SARS-CoV-2 infection activates brown adipose tissue and instigates a browning or beige phenotype of white adipose tissues, including augmented NST. This browning phenotype was also observed in post-mortem adipose tissue of four patients who died of COVID-19. Mechanistically, high levels of vascular endothelial growth factor (VEGF) in the adipose tissue induces adipose browning through vasculature–adipocyte interaction. Inhibition of VEGF blocks COVID-19-induced adipose tissue browning and NST and partially prevents infection-induced body weight loss. Our data suggest that the browning of adipose tissues induced by COVID-19 can contribute to adipose tissue atrophy and weight loss observed during infection. Inhibition of VEGF signaling may represent an effective approach for preventing and treating COVID-19-associated weight loss.

## Abstract (web summary)

Jing et al. show that COVID-19 infection causes white adipose tissue (AT) browning in mice and hamsters, which is mediated by VEGF action in the AT. VEGF blockade can ameliorate browning phenotype and COVID-19-induced weight loss, potentially providing a strategy to treat infection-induced AT atrophy.

## Main

**Fig. 1:**
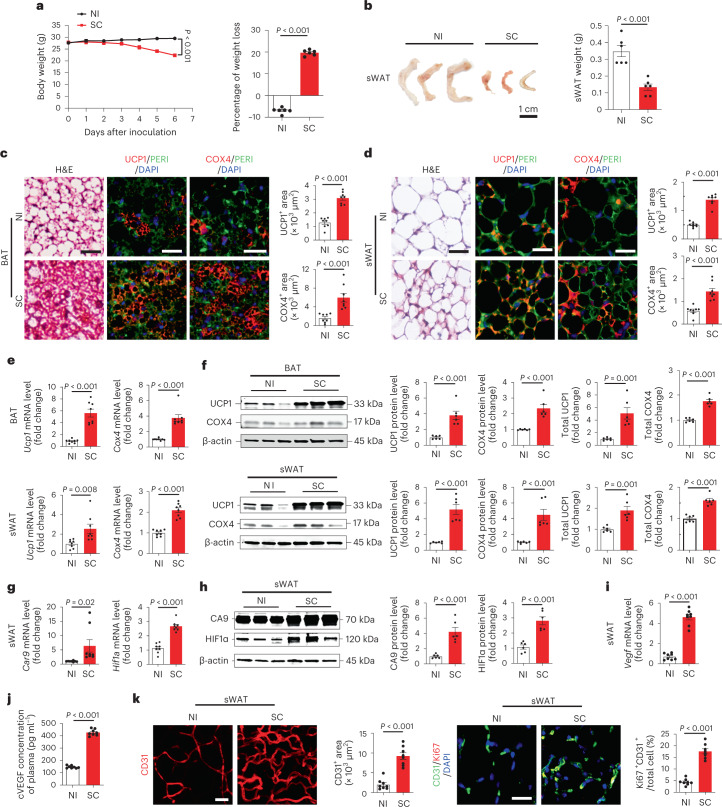
SARS-CoV-2 induces adipose atrophy in mice. **a**, Body weights of non-infected (NI) and day-6 post-SC-infected mice (*n* = 6 mice per group). **b**, Representative images of sWAT and quantification of adipose depot weights of NI- or day-6 post-SC-infected mice (*n* = 6 samples per group). **c**,**d**, Histological and immunohistochemical analyses of BAT and sWAT of NI or day-6 post-SC-infected mice by staining with hematoxylin and eosin (H&E), perilipin (PERI), UCP1 and COX4. Immunohistological sections were counterstained (blue) by 4,6-diamidino-2-phenylindole (DAPI). UCP1- and COX4-positive signals of BAT and sWAT were quantified (*n* = 8 random fields per group). **e**, mRNA levels of browning markers of *Ucp1* and *Cox4* in BAT and sWAT of NI or day-6 post-SC-infected mice were quantified by qPCR (*n* = 8 samples per group). **f**, Immunoblot analysis of UCP1 and COX4 protein levels of BAT and sWAT in NI or day-6 post-SC-infected mice. β-actin served as internal control. Quantification of relative and total amount of UCP1 and COX4 (*n* = 6 samples per group). **g**, Quantification of *Car9* and *Hif1a* mRNA levels by qPCR in sWAT of NI or day-6 post-SC-infected mice (*n* = 8 samples per group). **h**, Quantification of HIF1α and CA9 protein levels by immunoblot in sWAT of NI or day-6 post-SC-infected mice. β-actin served as internal controls (*n* = 6 samples per group). **i**, qPCR quantification of *Vegf* mRNA levels in sWAT of NI or day-6 post-SC-infected mice (*n* = 8 samples per group). **j**, Quantification of cVEGF levels in the plasma of NI or day-6 post-SC-infected mice (*n* = 8 mice per group). **k**, CD31 staining and quantification of microvessels in sWAT of NI or day-6 post-SC-infected mice. CD31 and Ki67 double-positive signals of sWAT were quantified (*n* = 8 random fields per group). Data are presented as mean ± s.e.m. Statistical analysis was performed using two-sided unpaired Student’s *t*-tests. SC, SARS-CoV-2. Scale bar, 50 μm. Source data

**Extended Data Fig. 1:**
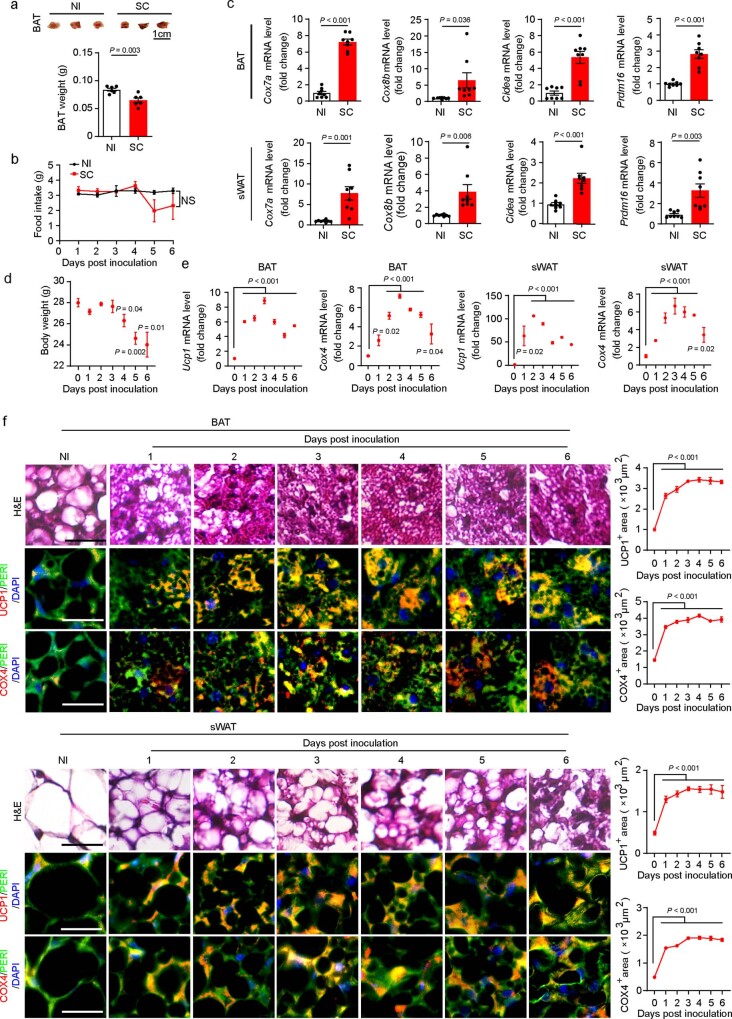
SARS-CoV-2 instigates adipose browning. **(a)** Representative images of BAT and quantification of brown adipose depot weights of NI- or day-6-post SC-infected mice (n = 6 samples per group). **(b)** Daily food intake of NI- and SC-infected mice (n = 6 mice per group). **(c)** mRNA levels of browning markers of *Cox7a, Cox8b, Cidea*, and *Prdm16* in BAT and sWAT of NI- or day-6-post SC-infected mice were quantified by qPCR (n = 8 samples per group). **(d)** Body weight of mice at various infected time points (n = 4 mice per group). **(e)** mRNA levels of browning markers of *Ucp1, Cox4* in BAT and sWAT of mice at various infected time points were quantified by qPCR (n = 4 samples per group). **(f)** Histological and immunohistochemical analyses of BAT and sWAT of mice at various infected time points by staining with H&E, perilipin (PERI), UCP1 and COX4. Immunohistological sections were counterstained by DAPI (blue). UCP1 and COX4 positive signals of BAT and sWAT were quantified (n = 8 random fields per group). Data are presented as means ± s.e.m. Statistical analysis was performed using two-sided unpaired t-tests. NS = not significant. SC = SARS-CoV-2. Scale bar = 50 μm. Source data

To study the mechanism underlying COVID-19-associated weight loss, a transgenic mouse model developed by knocking in human angiotensin-converting enzyme 2 (ACE2)^8^ was employed in our study. In this model, progressive weight loss alongside ‘wild-type’ SARS-CoV-2 virus infection was observed within a relatively short period, typically with significant weight loss within 1 week (Fig. 1a). Although variations existed among individual animals, approximately 20% weight loss as the ethical endpoint was observed within 2 weeks. Examination of adipose depots showed an approximately threefold reduction of subcutaneous white adipose tissue (WAT) (sWAT) (Fig. 1b). A slight decrease of brown adipose tissue (BAT) was also detected in SARS-CoV-2-infected animals (Extended Data Fig. 1a). Despite body weight loss, food intake was not significantly different between the non-infected and infected groups (Extended Data Fig. 1b).

Histological and immunohistochemical analyses of BAT demonstrated an activated phenotype with dense intracellular structures, high contents of COX4^+^ mitochondria and high expression levels of uncoupling protein 1 (UCP1) (Fig. 1c). Similarly, sWAT from SARS-CoV-2-infected mice exhibited a marked browning phenotype with smaller adipocyte sizes and morphologically appeared as multivesicular structures (Fig. 1d). Consistent with morphological changes, high contents of COX4^+^ mitochondria and UCP1^+^ structures also existed in sWAT adipocytes (Fig. 1d). Quantification of messenger RNA levels of adipocyte browning markers, including *Cox4*, *Ucp1*, *Cox7a*, *Cox8b*, *Cidea* and *Prdm16* validated browning phenotypes in both BAT and sWAT (Fig. 1e and Extended Data Fig. 1c). Quantitative analysis of UCP1 and COX4 protein levels further corroborated browning of BAT and sWAT (Fig. 1f). Decreases of lipid droplets in adipocytes of SARS-CoV-2-infected adipose depots might create a superficial browning phenotype of decreased adipocyte sizes and increases of expression browning proteins such as UCP1 and COX4. To exclude this possibility, we measure the total amount of UCP1 and COX4 proteins in the entire BAT and sWAT depots by a published immunoblot-based method^9^. Consistent with increased levels of mRNA expression, the total amount of UCP1 and COX4 in the entire BAT and sWAT depots was markedly increased in SARS-CoV-2-infected mice. Thus, SARS-CoV-2 infection augments adipose browning. Together, these data demonstrate that SARS-CoV-2 infection in mice augments a browning phenotype in BAT and sWAT.

**Extended Data Fig. 2:**
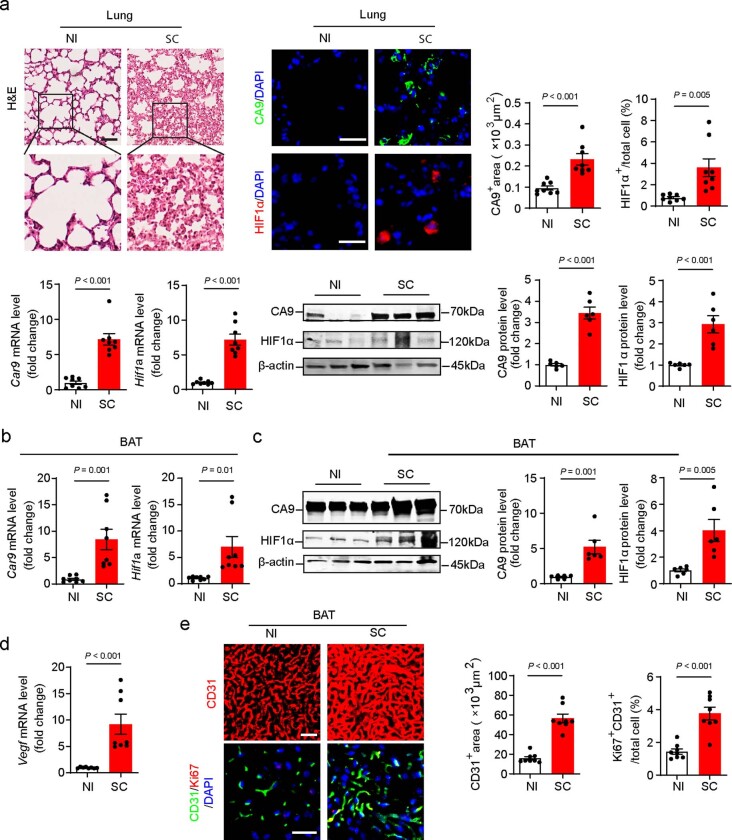
SARS-CoV-2 instigates tissue hypoxia and VEGF expression in mice. **(a)** Histological and immunohistochemical analyses of lung tissues of NI- and day-6-post SC-infected mice by staining with H&E, HIF1α and CA9. Tissue sections were counterstained with DAPI (blue). Quantification of HIF1α and CA9 positive signals in lung tissues (n = 8 random fields per group). Quantification of *Car9* and *Hif1a* mRNA and HIF1α and CA9 protein levels by qPCR (n = 8 samples per group) and Western immunoblot (n = 6 samples per group) in lung tissues. **(b)** Quantification of *Car9* and *Hif1a* mRNA levels by qPCR in BAT of NI- or day-6-post SC-infected mice (n = 8 samples per group). **(c)** Quantification of HIF1α and CA9 protein levels by Western immunoblot in BAT of NI- or day-6-post SC-infected mice. β-actin served as internal controls (n = 6 samples per group). **(d)** qPCR quantification of *Vegf* mRNA levels in BAT of NI- or day-6-post SARS-CoV-2-infected mice (n = 8 samples per group). **(e)** CD31 and Ki67 staining and quantification of microvessels in BAT of NI- or day-6-post SC-infected mice. Tissue sections were counterstained with DAPI (blue). CD31 and Ki67 double positive signals of BAT were quantified (n = 8 random fields per group). Data are presented as means ± s.e.m. Statistical analysis was performed using two-sided unpaired t-tests. NI = non-infected. SC = SARS-CoV-2. Scale bar = 50 μm. Source data

Lung tissues of SARS-CoV-2-infected mice were infiltrated with extravasated non-cellular and cellular components in the alveolar space, leading to severe hypoxia (Extended Data Fig. 2a). Accordingly, mRNA and protein levels of CA9 and HIF1α were markedly increased in the lung tissue of the SARS-CoV-2-infected animals (Extended Data Fig. 2a). Reconciling with local hypoxia in the lung tissue, sWAT also suffered from hypoxia by showing high mRNA levels of *Car9* and *Hif1a* expression (Fig. 1g). Immunoblots further validate the high levels of HIF1α and CA9 proteins in sWAT of SARS-CoV-2-infected mice (Fig. 1h).

Expression levels of VEGF, a main target of HIF1α^10,11^, in sWAT were markedly increased (Fig. 1i). Consequently, circulating VEGF (cVEGF) levels in the plasma of SARS-CoV-2-infected animals were markedly increased (Fig. 1j). In concordance with high VEGF levels, microvascular density in sWAT of SARS-CoV-2-infected mice was also markedly increased and some of these microvessels showed Ki67 positivity overlapping with CD31^+^ signals, indicating proliferating endothelial cells in angiogenic vessels (Fig. 1k). Similar to sWAT, BAT also experienced tissue hypoxia by expression of high levels of CA9 and HIF1a mRNAs and proteins (Extended Data Fig. 2b,c). Consequently, high VEGF expression and vascular density existed in BAT of SARS-CoV-2-infected animals (Extended Data Fig. 2d,e). These results show that SARS-CoV-2-infected adipose tissues contain high levels of VEGF and increased microvessels.

Significant body weight loss was detected after day 4 of SARS-CoV-2 infection and progressively decreased thereafter (Extended Data Fig. 1d). Notably, BAT became activated only 24 h after SARS-CoV-2 infection and intensified until day 3 (Extended Data Fig. 1e,f). Marked increases of mRNA and protein levels of UCP1 and COX4 were readily detectable 24 h after infection. Similar to BAT activation, sWAT also exhibited an overt browning phenotype by high expression of UCP1 and COX4 mRNA and protein 24 h after SARS-CoV-2 infection and became maximally activated after day 3 infection (Extended Data Fig. 1e,f). Additionally, visceral WAT (vWAT) browning occurred at similar time points as sWAT (Extended Data Fig. 5m,n). These data show that browning of various adipose depots occurs before body weight loss in the SARS-CoV-2-infected experimental model.

**Extended Data Fig. 3:**
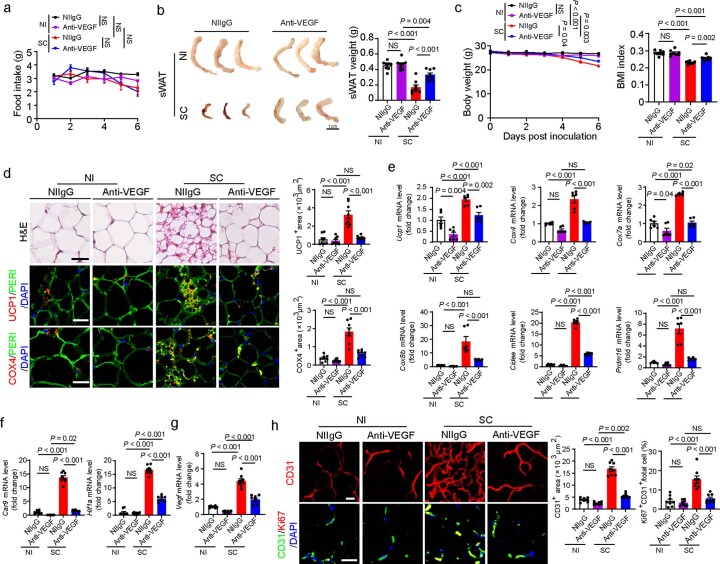
Blocking VEGF instigates adipose whitening and prevents body weight loss in mice. **(a)** Daily food intake of NI- and day-6-post SC-infected mice (n = 8 mice per group). Statistics on day 6 are presented. **(b)** Representative pictures of sWAT, and quantification of adipose tissue weights of non-immune IgG (NIIgG)- and anti-VEGF-treated NI- or day-6-post SC-infected mice (n = 8 samples per group). **(c)** Body weight and body mass index (BMI) of NIIgG- and anti-VEGF-treated NI- or day-6-post SC-infected mice (n = 8 mice per group). Statistics of body weight on day 6 are shown. **(d)** Histological and immunohistochemical analyses of sWAT of NIIgG- and anti-VEGF-treated NI- or day-6-post SC-infected mice by staining with H&E, Perilipin (PERI), UCP1 and COX4. Tissue sections were counter stained with DAPI (blue). Quantifications of UCP1 and COX4 positive signals in sWAT (n = 8 random fields per group). **(e)** mRNA levels of browning markers of *Ucp1, Cox4, Cox7a, Cox8b, Cidea*, and *Prdm16* in sWAT of NIIgG- and anti-VEGF-treated NI- or day-6-post SC-infected mice were quantified by qPCR (n = 6 samples per group). **(f)** qPCR quantification of *Hif1a* and *Car9* mRNA levels in sWAT of NIIgG- and anti-VEGF-treated NI- or day-6-post SC-infected mice (n = 8 samples per group). **(g)** qPCR quantification of *Vegf* mRNA levels in sWAT of NIIgG- and anti-VEGF-treated NI- or day-6-post SC-infected mice (n = 8 samples per group). **(h)** CD31 and Ki67 staining and quantification of microvessels in sWAT of NIIgG- and anti-VEGF-treated NI- or day-6-post SC-infected mice. Tissue sections were counterstained with DAPI (blue). CD31 and Ki67 positive signals of sWAT were quantified (n = 8 random fields per group). Data are presented as means ± s.e.m. Statistical analysis was performed using one-way analysis of variance (ANOVA) followed by Tukey multiple-comparison test. NS = not significant. NI = non-infected. SC = SARS-CoV-2. Scale bar = 50 μm. Source data

Previous work from our laboratory and others demonstrates that VEGF is a crucial angiogenic factor that augments a browning phenotype in adipose tissues^12–16^. To investigate the functional role of VEGF in adipose browning, we employed a loss-of-function experimental approach by using an anti-mouse neutralizing antibody (VEGF blockade)^17^. Under the standard mouse housing temperature of 22 °C ± 2 °C, anti-VEGF treatment did not alter food intake in the SARS-CoV-2-infected animals relative to controls (Extended Data Fig. 3a). Treatment of SARS-CoV-2-infected mice with VEGF blockade largely restored the sWAT mass relative to the non-immune IgG (NIIgG)-treated sWAT (Extended Data Fig. 3b). We should emphasize that VEGF blockade did not completely prevent the loss of sWAT weight. In contrast, VEGF blockade had no impact on tissue mass of sWAT in the non-infected healthy animals (Extended Data Fig. 3b). Consistent with restoration of sWAT, VEGF blockade also significantly increased total body weight and body mass index (BMI) of SARS-CoV-2-infected animals relative to the control group (Extended Data Fig. 3c).

**Extended Data Fig. 4:**
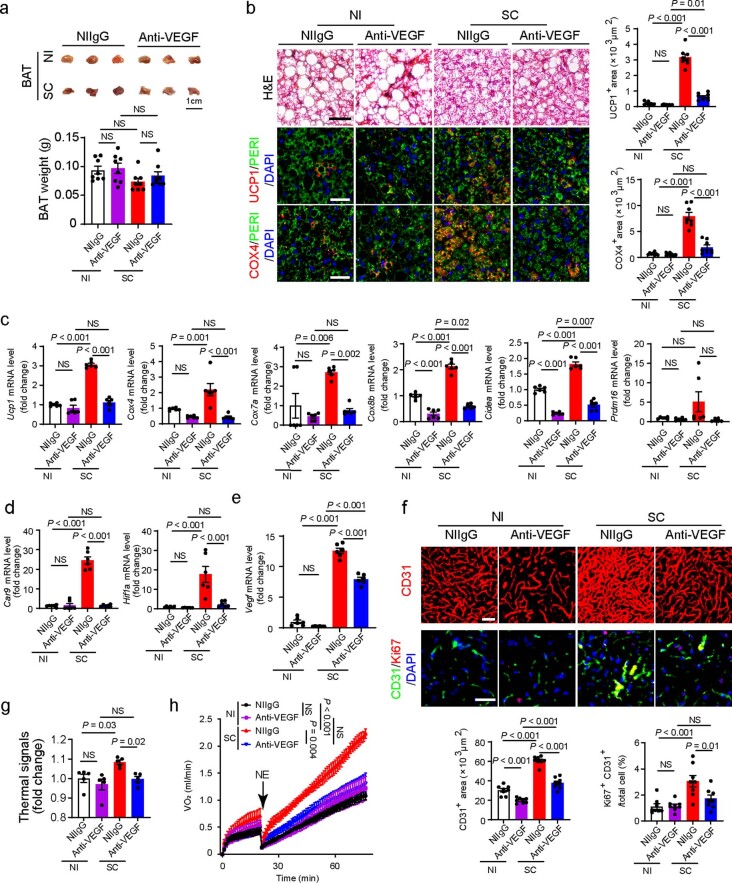
BAT whitening and ablation of NST by blocking VEGF. **(a)** Representative pictures of BAT and quantification of BAT weights of non-immune IgG (NIIgG)- and anti-VEGF-treated NI- or day-6-post SC-infected mice (n = 8 samples per group). **(b)** Histological and immunohistochemical analyses of BAT of NIIgG- and anti-VEGF-treated NI- or day-6-post SC-infected mice by staining with H&E, Perilipin (PERI), UCP1 and COX4. Tissue sections were counter stained with DAPI (blue). Quantifications of UCP1 and COX4 positive signals in BAT (n = 8 random fields per group). **(c)** mRNA levels of browning markers of *Ucp1, Cox4, Cox7a, Cox8b, Cidea*, and *Prdm16* in BAT of NIIgG- and anti-VEGF-treated NI- or day-6-post SC-infected mice were quantified by qPCR (n = 6 samples per group). **(d)** qPCR quantification of *Car9* and *Hif1a* mRNA levels in BAT of NIIgG- and anti-VEGF-treated NI- or day-6-post SC-infected mice (n = 6 samples per group). **(e)** qPCR quantification of *Vegf* mRNA levels in BAT of NIIgG- and anti-VEGF-treated NI- or day-6-post SC-infected mice (n = 6 samples per group). **(f)** CD31 and Ki67 staining and quantification of microvessels in BAT of NIIgG- and anti-VEGF-treated NI- or day-6-post SC-infected mice. Tissue sections were counterstained with DAPI (blue). CD31 and Ki67 double positive signals of BAT were quantified (n = 8 random fields per group). **(g)** Quantification of interscapular thermal signals of NIIgG- and anti-VEGF-treated NI- or day-3-post SC-infected mice (n = 5 mice per group). **(h)** Measurements of NST by norepinephrine in NIIgG- and anti-VEGF-treated NI- or day-3-post SC-infected mice. NE = norepinephrine (n = 5 mice per group). Data are presented as means ± s.e.m. Statistical analysis was performed using one-way analysis of variance (ANOVA) followed by Tukey multiple-comparison test. NS = not significant. NI = non-infected. SC = SARS-CoV-2. Scale bar = 50 μm. Source data

Histological and immunohistochemical examination of sWAT in SARS-CoV-2-infected mice showed a marked whitening phenotype by VEGF blockade, which was nearly indistinguishable from NIIgG-treated sWAT (Extended Data Fig. 3d). Consistent with morphological whitening, VEGF blockade-treated sWAT showed markedly mitigated levels of UCP1 and COX4 (Extended Data Fig. 3d,e). Additionally, quantitative analysis of mRNA levels of browning markers demonstrated marked decreases of *Cox4*, *Ucp1*, *Cox7a*, *Cox8b*, *Cidea* and *Prdm16* levels (Extended Data Fig. 3e). Along with sWAT whitening, VEGF blockade also alleviated adipose hypoxia by mitigating *Car9*, *Hif1a* and *Vegf* expression levels (Extended Data Fig. 3f,g). Consequently, anti-VEGF treatment also inhibited adipose angiogenesis in SARS-CoV-2-infected animals (Extended Data Fig. 3h). Similar whitening phenotypes of anti-VEGF-treated BAT were also observed (Extended Data Fig. 4a–f).

SARS-CoV-2 infection significantly increased thermal signals compared to those in non-infected animals (Extended Data Fig. 4g). VEGF blockade largely ablated the thermal effect in SARS-CoV-2-infected animals, even though anti-VEGF had no impact on thermogenesis in non-infected mice (Extended Data Fig. 4g). SARS-CoV-2 infection markedly increased NST in non-immune IgG-treated mice (Extended Data Fig. 4h). Of note, VEGF blockade ablated SARS-CoV-2-induced NST, which was indistinguishable from non-infected control mice (Extended Data Fig. 4h). Anti-VEGF treatment alone in non- SARS-CoV-2-infected mice had no effect on suppression of NST metabolism. Thus, our findings revealed a VEGF-dependent mechanism of adipose browning and NST metabolism in SARS-CoV-2-infected mice.

**Extended Data Fig. 5:**
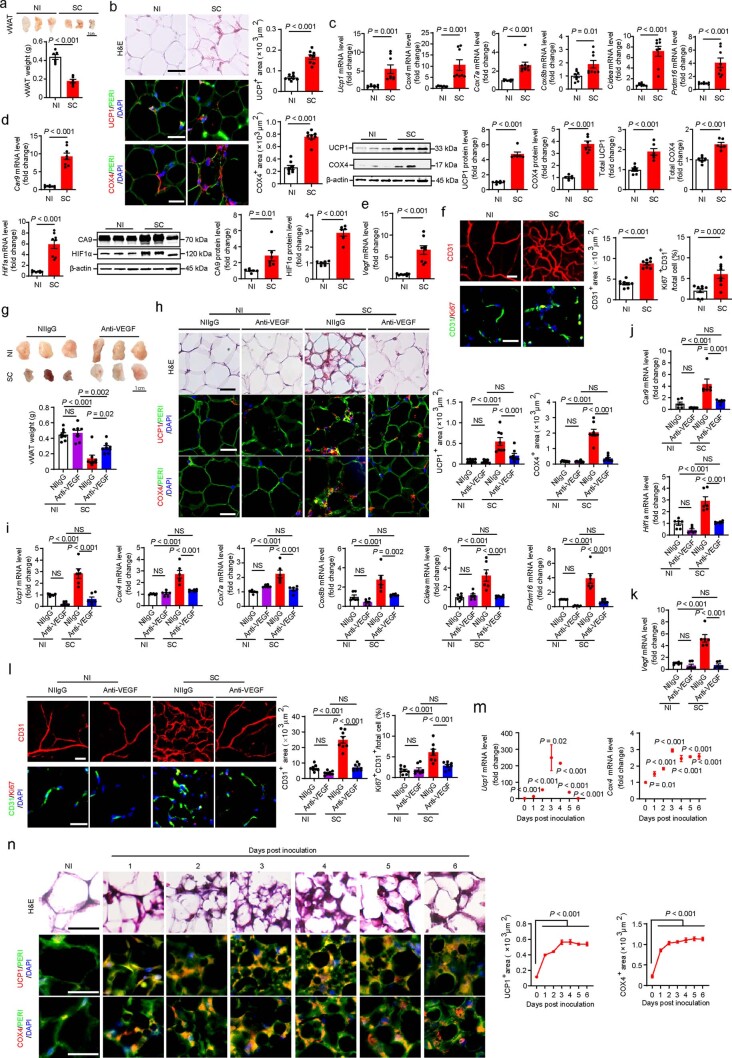
VEGF-dependent mechanisms of SARS-CoV-2-induced adipose browning. **(a)** Representative pictures and weight of vWAT and vWAT weight of NI- and day-6-post SC-infected mice (n = 6 samples per group). **(b)** Histological and immunohistochemical analyses of vWAT of NI- and day-6-post SC-infected mice by staining with H&E, Perilipin (PERI), UCP1 and COX4. Quantification of UCP1 and COX4 positive signals in vWAT (n = 8 random fields per group). **(c, d, e)** Quantification of *Ucp1, Cox4, Cox7a, Cox8b, Cidea, Prdm16, Car9, Hif1a* and *Vegf* mRNA level (n = 8 samples per group); UCP1, COX4, HIF1α and CA9 protein level in vWAT of NI- and day-6-post SC-infected (n = 6 samples per group). **(f)** CD31 and Ki67 staining and quantification of microvessels in vWAT of NI- or day-6-post SC-infected mice (n = 8 random fields per group). **(g)** Representative pictures of vWAT and vWAT weight of NIIgG- and anti-VEGF-treated NI- or day-6-post SC-infected mice (n = 8 samples per group). **(h)** Histological and immunohistochemical analyses of vWAT of NIIgG- and anti-VEGF-treated NI- or day-6-post SC-infected mice by staining with H&E, Perilipin (PERI), UCP1 and COX4. Quantifications of UCP1 and COX4 positive signals in vWAT (n = 8 random fields per group). **(i, j, k)** Quantification of *Ucp1, Cox4, Cox7a, Cox8b, Cidea, Prdm16, Car9, Hif1a* and *Vegf* mRNA levels in vWAT of NIIgG- and anti-VEGF-treated NI- or day-6-post SC-infected mice (n = 6 samples per group). **(l)** CD31 and Ki67 double staining and quantification of microvessels in vWAT of NIIgG- and anti-VEGF-treated NI- or day-6-post SC-infected mice (n = 8 random fields per group). **(m)** Quantification of *Ucp1* and *Cox4* mRNA levels in vWAT of SC-infected mice at various time points (n = 4 samples per group). **(n)** Histological and immunohistochemical analyses of vWAT in SC-infected mice at various time points by staining with H&E, perilipin (PERI), UCP1 and COX4. UCP1^+^ and COX4^+^ signals were quantified (n = 8 random fields per group). Data are presented as means ± s.e.m. Statistical analysis was performed using one-way analysis of variance (ANOVA) followed by Tukey multiple-comparison test and two-sided unpaired t-tests. NS = not significant. NI = non-infected. SC = SARS-CoV-2. Scale bar = 50 μm. Source data

To study whether SARS-CoV-2 infection also induced a browning phenotype of vWAT, we performed immunohistochemical and molecular analyses using similar experimental approaches for BAT and sWAT. Similar to sWAT, SARS-CoV-2 infection also augmented vWAT mass, a browning phenotype, exhibiting upregulation of UCP1, COX4 and other browning markers (Extended Data Fig. 5a–c). In vWAT of SARS-CoV-2-infected mice, marked increases of tissue hypoxia, HIF1α expression, VEGF, microvascular density and CD31^+^Ki67^+^ double-positive signals were detected (Extended Data Fig. 5d–f). Treatment of SARS-CoV-2-infected mice with VEGF blockade significantly prevented the loss of vWAT mass by whitening adipocytes (Extended Data Fig. 5g–l). These data indicate that SARS-CoV-2 augments vWAT browning through a VEGF-dependent mechanism and anti-VEGF therapy prevents adipose atrophy.

**Fig. 2:**
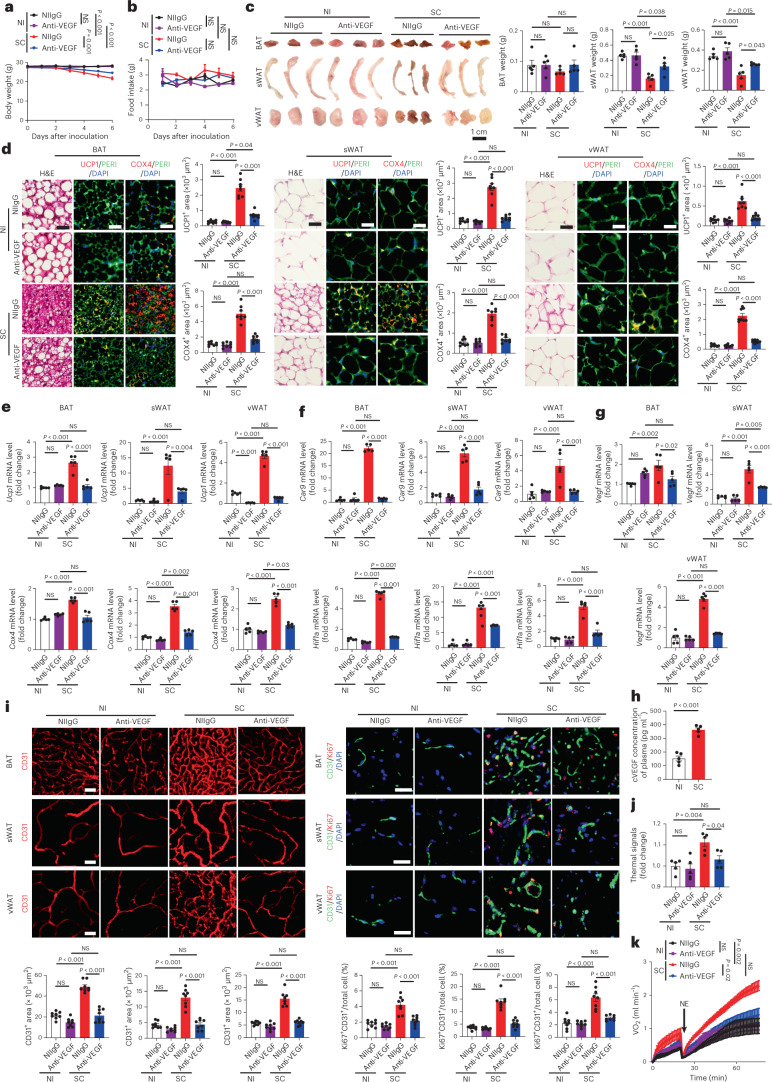
SARS-CoV-2 instigates adipose browning and NST metabolism under thermoneutrality. **a**, Body weights of non-immune IgG (NIIgG)- and anti-VEGF-treated NI or SC-infected mice (*n* = 5 mice per group). Statistics on day 6 are presented. **b**, Daily food intake of NIIgG- and anti-VEGF-treated NI or SC-infected mice (*n* = 5 mice per group). Statistics on day 6 are presented. **c**, Representative images of adipose depots of each group and quantification of adipose depot weights of NIIgG- and anti-VEGF-treated NI or day-6 post-SC-infected mice (*n* = 5 samples per group). **d**, Histological and immunohistochemical analyses of BAT, sWAT and vWAT of NIIgG- and anti-VEGF-treated NI or day-6 post-SC-infected mice by staining with H&E, PERI (green), UCP1 (red) and COX4 (red). Tissue sections were counterstained with DAPI (blue). Quantifications of UCP1- and COX4-positive signals (*n* = 8 random fields per group). **e**, mRNA levels of browning markers of *Ucp1* and *Cox4* of NIIgG- and anti-VEGF-treated NI or day-6 post-SC-infected mice were quantified by qPCR (*n* = 5 samples per group). **f**, qPCR quantification of *Car9* and *Hif1a* mRNA levels of NIIgG- and anti-VEGF-treated NI or day-6 post-SC-infected mice (*n* = 5 samples per group). **g**, qPCR quantification of *Vegf* mRNA levels of NIIgG- and anti-VEGF-treated NI or day-6 post-SC-infected mice (*n* = 5 samples per group). **h**, Quantification of circulating VEGF (cVEGF) levels in the plasma of NI or day-6 post-SC-infected mice (*n* = 5 mice per group). **i**, CD31 and Ki67 staining and quantification of microvessels of NI or day-6 post-SC-infected mice. Tissue sections were counterstained with DAPI. CD31 and Ki67 double-positive signals were quantified (*n* = 8 random fields per group). **j**, Quantification of interscapular thermal signals of NIIgG- and anti-VEGF-treated NI or day-3 post-SC-infected mice (*n* = 5 mice per group). **k**, Measurements of NST by norepinephrine in NIIgG- and anti-VEGF-treated NI or day-3 post-SC-infected mice. NE, norepinephrine (*n* = 5 mice per group). Data are presented as mean ± s.e.m. Statistical analysis was performed using one-way analysis of variance (ANOVA) followed by Tukey multiple-comparison test and two-sided unpaired Student’s *t*-tests. NS, not significant. Scale bar, 50 μm. Source data

To exclude the possibility of low temperature in contributing to adipose browning, we performed the same experiments of adipose browning, NST and anti-VEGF treatment under 30 °C thermoneutrality. Similar to 22 °C, SARS-CoV-2 infection augmented nearly identical browning phenotypes of BAT, sWAT and vWAT (Fig. 2a–e). Notably, UCP1 and COX4 were markedly increased in SARS-CoV-2-infected BAT, sWAT and vWAT (Fig. 2d,e). Again, VEGF blockade largely ablated the activation of browning of these adipose tissues, tissue hypoxia and VEGF expression (Fig. 2d–i). SARS-CoV-2 infection under thermoneutrality also markedly instigated thermal signals and NST metabolism, which were dependent on VEGF (Fig. 2j,k). Together, these data demonstrate that SARS-CoV-2 promotes adipose browning independently from cold exposure.

**Fig. 3:**
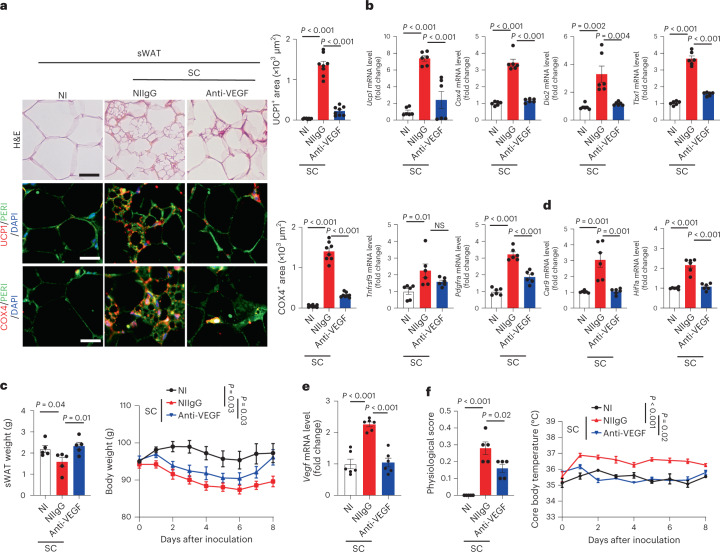
SARS-CoV-2 induces VEGF-dependent adipose browning in hamsters. **a**, Histological and immunohistochemical analyses of sWAT of NI hamsters and NIIgG- or anti-VEGF-treated day-8 post-SC-infected hamsters by staining with H&E, PERI, UCP1 and COX4. Tissue sections were counterstained with DAPI (blue). Quantification of UCP1- and COX4-positive signals in sWAT (*n* = 8 random fields per group). **b**, mRNA levels of browning markers of *Ucp1*, *Cox4*, *Dio2*, *Tbx1*, *Tnfrsf9* and *Pdgfra* in sWAT of NI hamsters and NIIgG- or anti-VEGF-treated day-8 post-SC-infected hamsters were quantified by qPCR (*n* = 6 samples per group). **c**, sWAT weight and body weight of NI hamsters and NIIgG- or anti-VEGF-treated day-8 post-SC-infected hamsters (*n* = 5 hamsters per group). Statistics are shown on day 8 of infection. **d**, qPCR quantification of *Car9* and *Hif1a* mRNA levels in sWAT of NI hamsters and NIIgG- or anti-VEGF-treated day-8 post-SC-infected hamsters (*n* = 6 samples per group). **e**, qPCR quantification of *Vegf* mRNA levels in sWAT of NI hamsters and NIIgG- or anti-VEGF-treated day-8 post-SC-infected hamsters (*n* = 6 samples per group). **f**, Physiological scores and core body temperature of NI hamsters and NIIgG- or anti-VEGF-treated day-8 post-SC-infected hamsters (*n* = 5 hamsters per group). Statistics are shown on day 8 of infection. Data are presented as mean ± s.e.m. Statistical analysis was performed using two-sided unpaired Student’s *t*-tests. Scale bar, 50 μm. Source data

**Extended Data Fig. 6:**
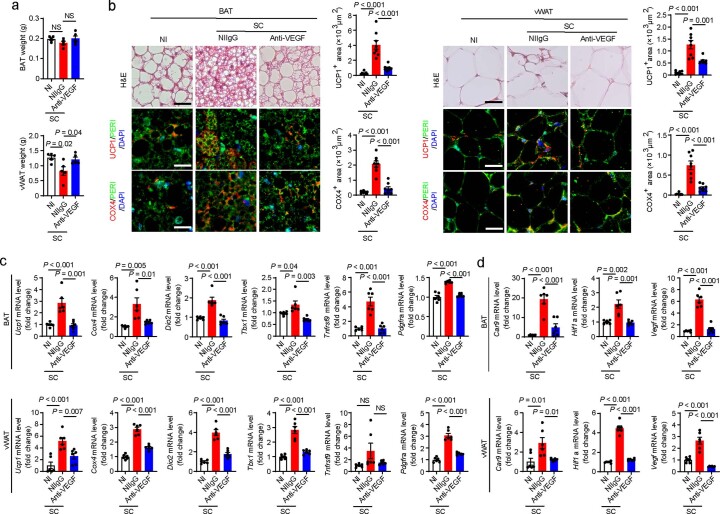
Suppression of BAT activation and vWAT browning by VEGF blockade in SARS-CoV-2-infected Syrian hamsters. **(a)** BAT and vWAT weights in NI- hamsters and NIIgG- or anti-VEGF-treated day-8-post SC-infected hamsters (n = 5 hamsters per group). **(b)** Histological and immunohistochemical analyses of BAT and vWAT of NI- hamsters and NIIgG- or anti-VEGF-treated day-8-post SC-infected hamsters by staining with H&E, Perilipin (PERI), UCP1 and COX4. Tissue sections were counterstained with DAPI (blue). Quantification of UCP1 and COX4 positive signals in BAT and vWAT (n = 8 random fields per group). **(c)** mRNA levels of browning markers of *Ucp1, Cox4, Dio2, Tbx1, Tnfrsf9*, and *Pdgfra* in BAT and vWAT of NI- hamsters and NIIgG- or anti-VEGF-treated day-8-post SC-infected hamsters were quantified by qPCR (n = 6 samples per group). **(d)** mRNA levels of *Car9, Hif1a* and *Vegf* in BAT and vWAT of NI- hamsters and NIIgG- or anti-VEGF-treated day-8-post SC-infected hamsters were quantified by qPCR (n = 6 samples per group). Data are presented as means ± s.e.m. Statistical analysis was performed using two-sided unpaired t-tests. NS = not significant. NI = non-infected. SC = SARS-CoV-2. Scale bar = 50 μm. Source data

To corroborate our findings in mice, we further investigated COVID-19-induced adipose browning in a Syrian hamster model^18^. After infection, Syrian hamsters exhibited similar pathologies as human COVID-19 pneumonia, including focal diffuse alveolar destruction in the infected lungs, hyaline member formation, inflammation response and fever^19^. Unlike the mouse model, SARS-CoV-2 causes pneumonia in non-genetically manipulated wild-type Syrian hamsters. Thus, the hamster model of COVID-19 is considered to be a clinically relevant animal model. Previous studies demonstrated that Syrian hamsters are hibernators during cold seasons and possess BAT and browning WATs^20^. As seen in mouse models, browning phenotypes of adipose tissues, expression of browning markers, including *Ucp1*, *Cox4*, *Dio2*, *Tbx1*, *Pdgfra* and *Tnfrsf9* expression and anti-VEGF responses were also observed in SARS-CoV-2-infected Syrian hamsters (Fig. 3 and Extended Data Fig. 6).

**Fig. 4:**
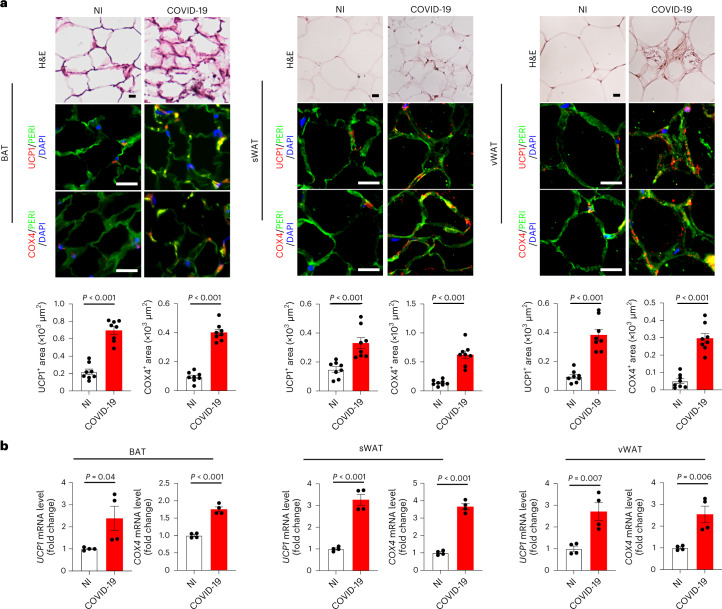
COVID-19 stimulates adipose browning in human patients with severe COVID-19. **a**, Histological and immunohistochemical analyses of sWAT, vWAT and supraclavicular BAT of autopsied fresh tissue samples from patients who died of COVID-19 infection. Non-infected patients who died of other diseases served as controls. Adipose tissues were stained with H&E, PERI, UCP1 and COX4. Tissue sections were counterstained with DAPI (blue). UCP1- and COX4-positive signals in sWAT, vWAT and BAT were quantified (*n* = 8 random fields per group). **b**, Quantification of mRNA levels of *UCP1* and *COX4* in human sWAT, vWAT and BAT (*n* = 4 samples per group). Data are presented as mean ± s.e.m. Statistical analysis was performed using two-sided unpaired Student’s *t*-tests. Scale bar, 50 μm. Source data

To relate our findings to clinical relevance, we studied the adipose tissues from human patients who died of severe COVID-19. A 61-year-old male patient without obvious comorbidity died of COVID-19 pneumonia. In addition, three other patients who died of COVID-19 were recruited to autopsy studies. Detailed demographic information, including age, sex and BMI of these patients was listed (Supplementary Table 1). Immunohistochemical staining showed that sWAT exhibited an overt browning phenotype by elevated expression of UCP1 and COX4 protein signals (Fig. 4a). Notably, vWAT also demonstrated increased expression of thermogenic protein of UCP1 and mitochondrial-specific protein COX4. These findings show that SARS-CoV-2 infection can augment a browning phenotype in visceral fat in humans. Previous studies in humans showed that BAT in adult humans is primarily located in the supraclavicular, paravertebral, mediastinal, para-aortic and suprarenal regions^5,21–23^. We, therefore, studied supraclavicular BAT from patients who died of severe COVID-19. Patients without COVID who died of other diseases served as controls in our experimental settings. Supraclavicular BAT from patients with COVID-19 exhibited high expression of UCP1 and COX4. These results from immunohistochemical analysis were further validated and quantified by qPCR, which showed marked increases of *UCP1* and *COX4* in adipose tissues (Fig. 4b). Together, these human data further corroborate the clinical relevance of our findings in preclinical models.

It is estimated that full activation of 1 g BAT in an adult human would burn away nearly 70 g WAT per year^5,6^. In rodents, both BAT activation and browning of sWAT by cold exposure, β3-adrenoceptor agonists and foods markedly contribute to NST^24^. Along with adipocyte browning, other cellular components, including microvasculature, preadipocytes and inflammatory cells in BAT and WAT undergo marked alterations^25^. Perhaps angiogenesis and vessel remodeling are the most overwhelming processes in browning adipose tissues^13^. Experimental evidence demonstrates that the VEGF–VEGFR2 signaling pathway plays a pivotal role in augmenting adipose angiogenesis. For example, pharmacological blockage of VEGF and genetic deletion of the *Vegfr2* gene in endothelial cells ablates cold-induced adipose browning and NST^12,15,16^.

Despite adipose atrophy in our experimental settings, we did not find muscular atrophy and liver weight reduction in SARS-CoV-2-infected animals versus non-infected control animals. Adipose atrophy may partly contribute to total body weight loss but is not fully correlated with total body weight loss. Perhaps other factors such as fever-related dehydration may also contribute to body weight loss.

**Extended Data Fig. 7:**
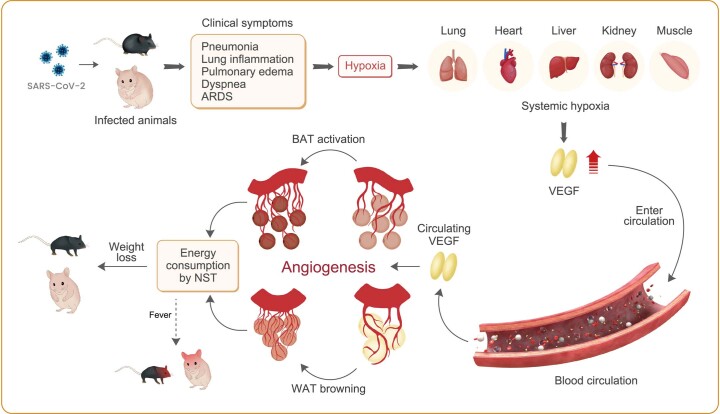
Schematic illustration of mechanisms underlying SARS-CoV-2-induced adipose atrophy and weight loss. SARS-CoV-2 infection triggers dyspnea and pathological changes in the infected lung tissues, including pulmonary edema, inflammation, and obstruction of pulmonary alveoli. These pathological alterations induce local hypoxia in the lung tissue and systemic hypoxia in other tissues. Hypoxia serves as a potent factor to induce VEGF expression through the HIF1α-VEGF promoter dependent mechanism. Non-heparin-binding smaller isoforms of VEGF enters the circulation and produces systemic effects on multiple tissues and organs. In the adipose tissue, the local and circulating VEGF promotes browning phenotypes of WATs and BAT. Activation of BAT and browning of WATs augment non-shivering thermogenesis (NST) by heat production, which may partly explain the SARS-CoV-2-induced fever. NST activation by SARS-CoV-2 also markedly augments energy consumption, leading to a lean phenotype and clinically manifesting as adipose atrophy and weight loss.

On the basis of our discoveries, we have hypothesized that high levels of VEGF in SARS-CoV-2-infected individuals may contribute to adipose browning and NST and blocking of VEGF may provide a therapeutic approach for preventing adipose atrophy and weight loss (Extended Data Fig. 7). In both mouse and hamster COVID-19 models, VEGF blockade markedly inhibited browning of WATs, indicating the VEGF-dependent mechanism of WAT browning. Although suppression of adipose browning by anti-VEGF treatment is difficult to be validated in human patients, histological and immunohistochemical analyses of autopsied human adipose tissue from patients who died of COVID-19 showed the existence of a browning phenotype. Thus, our findings are clinically relevant.

VEGF is a key upregulated growth factor caused by hypoxia, which displays potent angiogenic and vascular permeability effects^26,27^. VEGF has been reported to cause a brown-like phenotype of WATs^12,14–16,28–30^. In preclinical models, treatment of SARS-CoV-2-infected animals with VEGF blockade significantly prevented body weight loss by restoring WAT. Although SARS-CoV-2-infected animals suffer from acute weight loss and are different from human patients, the anti-cachexic effect of anti-VEGF treatment inexorably corroborates this therapeutic concept.

Another notable issue related to adipose browning and NST is fever, which is one of the most common and characteristic clinical symptoms of infectious diseases, including COVID-19 pneumonia; however, the molecular mechanisms underlying fever and heat production in the body remain elusive. In particular, the role of NST by browning adipose tissue in the development of fever and adipose atrophy symptoms during COVID-19 pneumonia is completely unknown. In this study, we provide evidence in mouse and hamster COVID-19 models that anti-VEGF may mitigate fever. Although the detailed mechanisms underlying the antipyretic effect are not completely understood at the time of writing, we reasonably speculate that whitening of WATs provides an attractive mechanism of the antipyretic effect of anti-VEGF drugs. Consistent with this notion, clinical trials with bevacizumab for treating patients with severe COVID-19 demonstrated that anti-VEGF therapy produces a potent antipyretic effect in nearly 100% of patients^31^.

We propose a therapeutic concept of treating COVID-19 weight loss by whitening adipose tissue. Anti-VEGF drugs provide a successful example of this type of therapy. Although we provide the proof-of-concept example for treating COVID-19, the same therapeutic principle can be probably expanded for treating other pulmonary diseases; however, we admit that differences exist between preclinical models in our study and human patients. Several factors, including genetic background, age, sex, comorbidity and body weight loss are intrinsically different between SARS-CoV-2 -infected humans and experimental animals, highlighting the importance of confirming our findings in humans.

## Methods

### Ethical permission for animal and human studies

All animals were performed in strict compliance with approved ethical permits, including 8298–2020 and 10513–2020. Animals were kept at the Astrid Fagraeus laboratory and the experiments were performed in a biosafety level 3 laboratory at the Karolinska Institutet under the guidelines of the Swedish Board of Agriculture. The biological clock cycle for animals was 12 h light–dark. Food for hamsters (Tiny Friends Farm, 1240789) and mice (Special Diets Services, CRM, 801722) was given freely. The protocols were approved by the local ethics committee, Stockholms Norra Djurförsöksetiska Nämnd and in compliance with the Animal Research Reporting of In Vivo Experiments guidelines. All animals were randomly divided into each group for exposure to 30 °C or room temperature and received various treatments. For human studies, all autopsies were conducted at the risk-autopsy facility of the Department of Clinical Pathology/Cytology, Karolinska University Hospital. All individuals were referred to the pathology department for the clinical autopsy to establish the precise cause of death. The study was approved by the Swedish Ethical Review Authority under approval nos. DNR 2020-02446 and 2020-04339.

### Animal COVID-19 models and drug treatment

Heterozygous K18-hACE C57BL/6J mice (strain 2B6.Cg-Tg(K18-ACE2) 2Prlmn/J) were purchased from the Jackson Laboratory (strain 034860). Animals were housed in a group of fewer than five animals per cage and fed with a standard chow diet. Mice at 12 weeks old were intranasally administered with ‘wild-type’ SARS-CoV-2 virus at the dose of 1 × 10^2^ plaque-forming units (p.f.u.) per mouse. SARS-CoV-2 viruses were isolated and expanded from clinical samples of wild-type SARS-CoV-2 obtained from G. McInerney’s group at the Karolinska Institute according to previously published protocols^32^. A rabbit anti-mouse VEGF neutralizing antibody at a dose of 7.5 mg kg^−1^ (BD0801, Simcere Pharmaceutical Company) was intraperitoneally injected into each mouse every other day, starting at day 0 of SARS-CoV-2 infection. NIIgG-antibody-treated animals served as controls using the same treatment regimen. All animal experiments were terminated using a lethal dose of isoflurane. Syrian hamsters at 8 weeks old were purchased from Janvier Labs. SARS-CoV-2 viral particles at the dose of 1 × 10^6^ p.f.u. were intranasally administrated into each hamster. Anti-VEGF and NIIgG treatment regimens, schedules and protocols were the same as those used in mouse studies.

### Measurements of food intake, body weight and BMI

To measure food intake and body weight, mice were caged two mice per cage and free-fed with normal chow. Food consumption and body weight of SARS-CoV-2-infected and non-infected mice were measured on a daily basis. BMI was calculated daily according to a previously published protocol^33^ by (body weight (g) / (crown − rump length (cm))^2^).

### H&E histological staining

Inguinal WAT was used as subcutaneous WAT in mice and hamsters. Epididymal WAT was used as vWAT in this study. Interscapular BAT was used as BAT. In humans, supraclavicular adipose is a commonly recognized BAT; sWAT was obtained under the skin in the abdominal region and vWAT was obtained from the mesentery adipose tissue. Paraffin-embedded tissues were cut into 5-μm-thick sections, de-paraffinized in Tissue-Clear (1466, Sakura) and rehydrated with sequential incubation in 99–95–70% ethanol using a stepwise procedure. Tissue slides were stained with hematoxylin (6765009, Thermo Fisher Scientific), followed by eosin (HT110116, Sigma). After dehydration with 95–99% ethanol, slides were mounted with a Pertex mounting medium (00801, HistoLab). Stained tissues were photographed using a light microscope (×20, Nikon Eclipse TS100) equipped with a camera (DS-Fi1, Nikon) and software (NIS-Elements F3.2).

### Immunohistochemistry

Paraffin-embedded tissues were cut into 5-μm-thick sections. Before staining, tissue slides were de-paraffinized by Tissue-Clear (1466, Sakura) and rehydrated with sequential incubation with 99–95–70% ethanol. Tissue sections were boiled for 20 min in an unmasking solution (H3300, VECTOR) and subsequently blocked with 4% serum. Tissue slides were stained overnight at 4 °C with primary antibodies against COX4 (1:300 dilution, NB110-39115, Novus Biologicals), UCP1 (1:300 dilution, PA1-24894, Thermo Fisher Scientific), Perilipin (1:300 dilution, NB100-60554, Novus Biologicals), CA9 (1:300 dilution, NB100-417, Novus Biologicals), HIF1α (1:300 dilution, 36169, Cell Signaling Technology), CD31 (1:300 dilution, 553370, BD Pharmingen) and Ki67 (1:300 dilution, PA5-19462, Thermo Fisher Scientific), followed by further staining for 1 h at room temperature with species-matched secondary antibodies: Alexa Fluor 555-labeled goat anti-rat antibody (1:300 dilution, A21434, Thermo Fisher Scientific), Alexa Fluor 488-labeled donkey anti-rat antibody (1:300 dilution, A21208, Thermo Fisher Scientific), Alexa Fluor 555 donkey anti-rabbit antibody (1:300 dilution, A31572, Thermo Fisher Scientific) and Alexa Fluor 488 donkey anti-goat antibody (1:300 dilution, A11055, Thermo Fisher Scientific). Positive signals were detected using a fluorescence microscope equipped with a camera (Nikon, DS-QilMC). Images were analyzed using an Adobe Photoshop software (CS6, Adobe) program.

### Whole-mount staining

Tissue samples were collected and fixed overnight with 4% PFA. Samples were cut into thin pieces and digested for 5 min with 20 mM proteinase K in a 10 mM Tris-buffer (pH 7.5), followed by incubation with 100% methanol for 30 min. Samples were incubated at 4 °C with 0.3% Triton X-100 PBS containing 3% skim milk overnight. Samples were washed with PBS five times, followed by incubation with a combination of a rat anti-mouse CD31 (1:300 dilution, 553370, BD Pharmingen) antibody overnight. Samples were incubated with an Alexa Fluor 555-labeled goat anti-rat secondary antibody (1:300 dilution, A21434, Thermo Fisher Scientific). After washing with PBS, tissues were mounted by a VECTASHIELD mounting medium (H1000, Vector Laboratories). Images were obtained by confocal microscopy (Nikon C1 confocal microscope, Nikon). Dimensional images of vessels were analyzed. Positive signals of CD31 area were calculated using Adobe Photoshop software (CS6, Adobe).

### RNA isolation and qPCR

Total RNAs were extracted from tissues using TRIzol (15-596-018, Invitrogen) and the GeneJET RNA purification kit (K0731, Thermo Fisher Scientific) according to the manufacturer’s protocol. Total RNA concentrations were determined by NanoDrop2000. cDNA was synthesized with the RevertAid cDNA synthesis kit (K1622, Thermo Fisher Scientific) and was subsequently used for qPCR analysis with a Power SYBR Green Master Mix (43-676-59, Invitrogen) using the StepOnePlus Real-Time PCR System. qPCR data were quantified from the threshold cycle (Ct) and relative expression levels were calculated by using the 2^−ΔΔCt^ method. All primers used are listed in Supplementary Table 2.

### Immunoblotting

Total proteins from adipose tissues were extracted by a RIPA lysis/extraction buffer (89900, Thermo Fisher Scientific) containing a Halt Protease Inhibitor Cocktail (87785, Thermo Fisher Scientific). An equal amount of protein from each sample was loaded onto Mini-PROTEAN TGX Gels (4561086, Bio-Rad) and transferred to PVDF membranes (1620184, Bio-Rad). Immunoblotting was incubated with the primary antibodies specific for UCP1 (1:1,000 dilution, PA1-24894, Thermo Fisher Scientific), COX4 (1:1,000 dilution, NB110-39115, Novus Biologicals), CA9 (1:1,000 dilution; NB100-417; Novus Biologicals) and HIF1α (1:1,000 dilution, 36169, Cell Signaling Technology). A primary antibody against β-actin (1:2,000 dilution, 3700, Cell Signaling Technology) was used to justify the sample loading levels. Secondary antibodies-conjugated with IRDye 680RD donkey anti-mouse (1:15,000 dilution, 926-68072, LI-COR Biosciences) and IRDye 800CW donkey anti-rabbit (1:15,000 dilution, 926-32213, LI-COR Biosciences) were incubated. Densitometry analysis was performed using the Odyssey CLX Imaging System (LI-COR Biosciences).

### ELISA

ELISA was used for determining mouse VEGF protein concentrations. To detect circulating protein levels, mouse plasma VEGF (MMV00, R&D Systems) were determined according to the manufacturer’s protocol with the standard curve. Absorbance values were detected at 450 nm using a microplate reader and values were calculated using the formula obtained from the trendline.

### Quantification of total amounts of UCP1 and COX4 protein

Determination of relative total amounts of proteins was performed using a previously published method^9^. In brief, a total volume of 200 μl homogenates from each adipose depot was prepared. Total protein contents of tissue homogenates were determined by the bicinchoninic acid assay. Each homogenate in 30 μg was loaded for immunoblot analysis. Positive signals of UCP1 and COX4 proteins were defined per unit in each sample. Each unit was multiplied by the amount of total protein in the same sample to obtain the total amount of protein.

### Infrared thermal imaging

According to our recently published method^34^, SARS-CoV-2-infected and non-infected mice under various experimental conditions were anesthetized and placed in the same position, followed by imaging on the back side using a thermal camera (Infrared Thermal Imager, Fotric, 346). Thermal images of each experimental mouse were analyzed by Fotric AnalyzIR software. Heat signals were quantified from images (*n* = 5 animals per group).

### Metabolic analyses

Owing to the strict regulation of COVID-19 work at our institution, oxygen consumption was indirectly measured using an oxygen detector (Oxygen and Carbon Dioxide Detector CM-505; cat. no. CM-505). NST measurements were performed using norepinephrine according to our previously published method^15^. Mice were anesthetized and O_2_ concentrations were measured by collecting samples every 1 min. The basal metabolic rate was measured before norepinephrine injection.

### Statistical analysis

Collected data analyses were performed using GraphPad Prism (GraphPad) and Microsoft 365 Excel. Data were presented as mean ± s.e.m. and statistical computations were performed using a standard two-tailed Student’s *t*-test and one-way ANOVA. *P* < 0.05 was deemed to be statistically significant.

### Reporting summary

Further information on research design is available in the Nature Portfolio Reporting Summary linked to this article.

## Supplementary information

Supplementary Information Supplementary Tables 1 and 2 and Supplementary Fig. 1

Reporting Summary

## Data Availability

Full scans of all immunoblots are provided in the Supplementary Information. Source data are provided with this paper.

## Source data

Source Data Fig. 1 Statistical Source Data.
Source Data Fig. 2 Statistical Source Data.
Source Data Fig. 3 Statistical Source Data.
Source Data Fig. 4 Statistical Source Data.
Source Data Extended Data Fig. 1 Statistical Source Data.
Source Data Extended Data Fig. 2 Statistical Source Data.
Source Data Extended Data Fig. 3 Statistical Source Data.
Source Data Extended Data Fig. 4 Statistical Source Data.
Source Data Extended Data Fig. 5 Statistical Source Data.
Source Data Extended Data Fig. 6 Statistical Source Data.
