# Supplementary material for: COVID-19 instigates adipose browning and atrophy through VEGF in small mammals
Source: Nat Metab. 2022 Dec 8;4(12):1674–83. doi: 10.1038/s42255-022-00697-4 (PMC9771808; doi:10.1038/s42255-022-00697-4)

nature metabolism

Letter

https://doi.org/10.1038/s42255-022-00697-4

COVID-19 instigates adipose browning and atrophy through VEGF in small mammals

In the format provided by the authors and unedited

COVID-19 instigates adipose browning and atrophy through VEGF in small mammals

**Supplementary Table 1**	Clinical characteristics
**Supplementary Table 2**	Primer sequences
**Supplementary Fig. 1**	Raw data of gels for immunoblots

Supplementary Table 1. Clinical characteristics

Groups	Age (years)	Sex	BMI (kg/m^2)
**Non-infected**			
Patient 1	69	Male	34.5
Patient 2	74	Male	35.5
Patient 3	75	Female	26.9
Patient 4	61	Female	24.9
**Covid-19**			
Patient 1	61	Male	27
Patient 2	55	Male	24.7
Patient 3	81	Female	26.9
Patient 4	57	Female	33.1

Supplementary Table 2. Primer sequences

Gene	Species	Forward primer sequence	Reverse primer sequence
Actin	mouse	5’-AGGCCCAGAGCAAGAGAGG-3’	5’-TACATGGCTGGGGTGTTGAA-3’
Ucp1	mouse	5’-AAACAGAAGGATTGCCGAAA-3’	5’-TGCATTCTGACCTTCACGAC-3’
Cox4	mouse	5’-CTATGTGTATGGCCCCATCC-3’	5’-AGCGGGCTCTCTCACTTCTTC-3’
Cox7a	mouse	5’-CAGCGTCATGGTCAGTCTGT-3’	5’-AGAAAACCGTGTGGCAGAGA-3’
Cox8b	mouse	5’-GAACCATGAAGCCAACGACT-3’	5’-GCGAAGTTCACAGTGGTTCC-3’
Cidea	mouse	5’-TGCTCTTCTGTATCGCCCAGT-3’	5’-TGCTCTTCTGTATCGCCCAGT-3’
Prdm16	mouse	5’-CAGCACGGTGAAGCCATTC-3’	5’-GCGTGCATCCGCTTGTG-3’
Car9	mouse/ hamster	5’-CTGAAGACAGGATGGAGAAG-3’	5’-GCAGAGTGCGGCAGAATG-3’
Hif1α	mouse/ hamster	5’-GTCGGACAGCCTCACCAAACAG-3’	5’-TAGGTAGTGAGCCACCAGTGTCC-3’
Vegf	mouse/ hamster	5’-ATCCGCATGATCTGCATGG-3’	5’-AGTCCCATGAAGTGATCAAGTTCA-3’
Actin	hamster	5’-AAGTGTGACGTCGACATCCG-3’	5’-GATCCACACAGAGTACTTGC-3’
Ucp1	hamster	5’-GAGCTGGTAACATATGACCT-3’	5’-TGTCCTGGCAGAGAGTTGAT-3’
Cox4	hamster	5’-AACAGGAGCACCAATGAGTG-3’	5’-TTGGCCTTCATGTCCAGCAT-3’
Dio2	hamster	5’-TACGGGGTAGCTTTTGAGCG-3’	5’-CTCCGCACTTCTTGGAGGTT-3’
Tbx1	hamster	5’-GGCGGGCAGGCGGATGTTC-3’	5’-TGTCATCCACAGGCACAAAG-3’
Tnfrsf9	hamster	5’-ATCTGCAGAGTGTGCGAAGG-3’	5’-TAACTCTTGACCCGGCTTGC-3’
Pdgfra	hamster	5’-GTGGGTATCGGATGGCCAAA-3’	5’-TTGTACTGTCCAGGCAGCAG-3’
ACTIN	human	5’-ATTGCCGACAGGATGCAGAA-3’	5’-GCTGATCCACATCTGCTGGAA-3’
UCP1	human	5’-CAACCCACAGAGGTCGTGAA-3’	5’-CCCGTGTAGCGAGGTTTGAT-3’
COX4	human	5’-CAGGGTATTTAGCCTAGTTGGC-3’	5’-GCCGATCCATATAAGCTGGGA-3’
COX7a	human	5’-TTCACGCAGAAGTTGGCAGG-3’	5’-GAGGTCAGTTTAGTTGGTGTGG-3’

Supplementary Figure 1

Raw data of gels for immunoblots.

Whole gel images. Boxes imply area shown in the figure.

Full scans of immunoblots in Fig. 1f

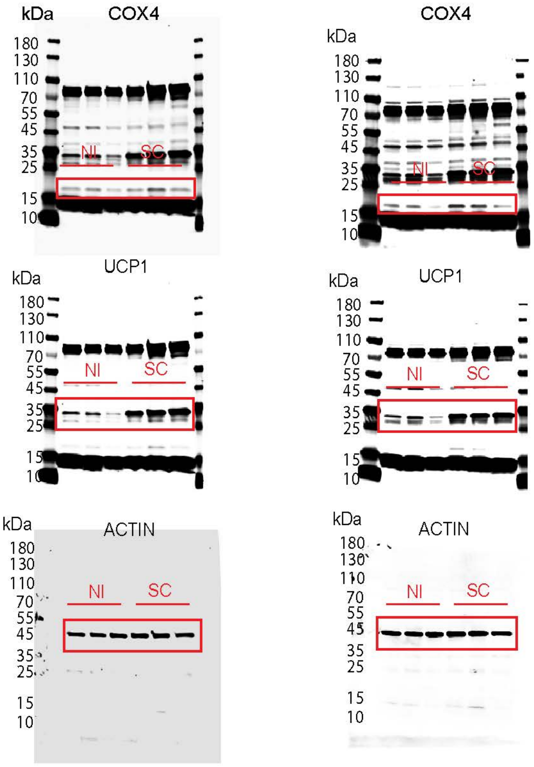

Full scans of immunoblots in Fig. 1h

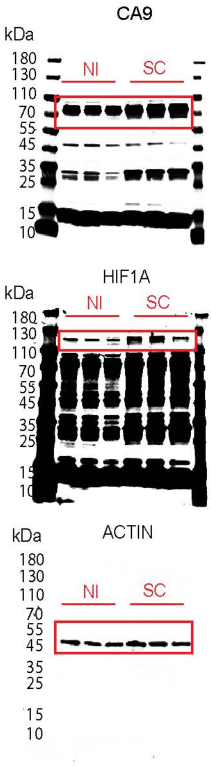

Supplementary Figure 1

Raw data of gels for immunoblots.

Whole gel images. Boxes imply area shown in the figure.

Full scans of immunoblots in Extended Data Fig. 2a and 2c

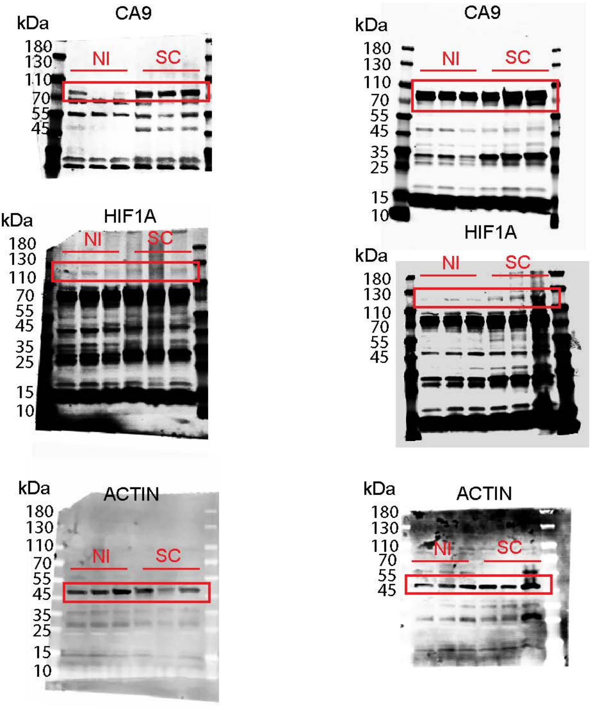

Supplementary Figure 1

Raw data of gels for immunoblots.

Whole gel images. Boxes imply area shown in the figure.

Full scans of immunoblots in Extended Data Fig. 5c and 5d

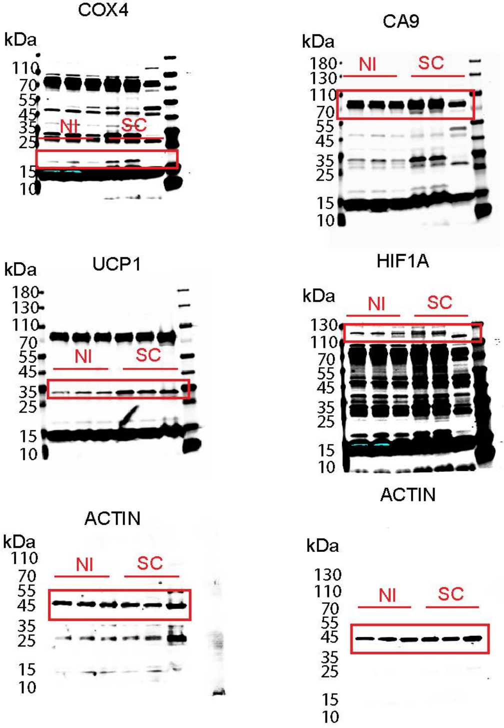

Supplement: Supplementary file 1 — Supplementary Tables 1 and 2 and Supplementary Fig. 1 [file 42255_2022_697_MOESM1_ESM.pdf]
