# Supplementary material for: COVID-19 instigates adipose browning and atrophy through VEGF in small mammals
Source: Nat Metab. 2022 Dec 8;4(12):1674–83. doi: 10.1038/s42255-022-00697-4 (PMC9771808; doi:10.1038/s42255-022-00697-4)
Supplement: Supplementary file 2 — Reporting Summary [file 42255_2022_697_MOESM2_ESM.pdf]

nature portfolio

Corresponding author(s): Yihai Cao

Last updated by author(s): Sep 28, 2022

Reporting Summary

Nature Portfolio wishes to improve the reproducibility of the work that we publish. This form provides structure for consistency and transparency in reporting. For further information on Nature Portfolio policies, see our Editorial Policies and the Editorial Policy Checklist.

Statistics

For all statistical analyses, confirm that the following items are present in the figure legend, table legend, main text, or Methods section.

n/a	Confirmed	
☐	☒	The exact sample size (*n*) for each experimental group/condition, given as a discrete number and unit of measurement
☐	☒	A statement on whether measurements were taken from distinct samples or whether the same sample was measured repeatedly
☐	☒	The statistical test(s) used AND whether they are one- or two-sided<br>*Only common tests should be described solely by name; describe more complex techniques in the Methods section.*
☐	☒	A description of all covariates tested
☐	☒	A description of any assumptions or corrections, such as tests of normality and adjustment for multiple comparisons
☐	☒	A full description of the statistical parameters including central tendency (e.g. means) or other basic estimates (e.g. regression coefficient) AND variation (e.g. standard deviation) or associated estimates of uncertainty (e.g. confidence intervals)
☒	☐	For null hypothesis testing, the test statistic (e.g. *F*, *t*, *r*) with confidence intervals, effect sizes, degrees of freedom and *P* value noted<br>*Give P values as exact values whenever suitable.*
☒	☐	For Bayesian analysis, information on the choice of priors and Markov chain Monte Carlo settings
☒	☐	For hierarchical and complex designs, identification of the appropriate level for tests and full reporting of outcomes
☒	☐	Estimates of effect sizes (e.g. Cohen's *d*, Pearson's *r*), indicating how they were calculated

Our web collection on statistics for biologists contains articles on many of the points above.

Software and code

Policy information about availability of computer code

Data collection	-Immunofluorescence images were captured using a camera (Nikon, DS-QilMC) with NIS-Elements D3.2 (Nikon) software and quantified using Adobe Photoshop software CS6 extended (Adobe) .<br>- The confocal images were recorded using the camera of a Nikon C1 confocal microscope with EZ-C1 3.91 software (Nikon) and quantified using Adobe Photoshop software CS6 extended (Adobe) .<br>- For H&E staining, the image was captured by a light microscope (Nikon Eclipse TS100) with the camera (DS-Fil, Nikon) using NIS-Elements F 3.2 software (Nikon), and quantified using Adobe Photoshop software CS6 extended (Adobe) .<br>-Immunoblots files were collected using an Odyssey CLx system (LI-COR) with an Image Studio version 3.1 (LI-COR).<br>-The oxygen consumption data were collected using Oxygen and Carbon Dioxide Detector (CM-505) .<br>-Thermal images captured by infrared thermal imager were quantified using Fotric AnalyzIR software.<br>-RT-PCR files were collected using a StepOnePlus™ Real-Time PCR System (Applied Biosystems).
Data analysis	Adobe Photoshop software CS6 extended (Adobe) program; Microsoft 365 Excel; GraphPad Prism 9.2.0(GraphPad Software); Image Studio ver 3.1; NIS-Elements F3.2; Fotric AnalyzIR software

For manuscripts utilizing custom algorithms or software that are central to the research but not yet described in published literature, software must be made available to editors and reviewers. We strongly encourage code deposition in a community repository (e.g. GitHub). See the Nature Portfolio guidelines for submitting code & software for further information.

Data

Policy information about availability of data

All manuscripts must include a data availability statement. This statement should provide the following information, where applicable:

- Accession codes, unique identifiers, or web links for publicly available datasets
- A description of any restrictions on data availability
- For clinical datasets or third party data, please ensure that the statement adheres to our policy

Full scans of all immunoblots are provided in the Supplementary Information. In addition, source data for all experiments are provided in this paper.

Human research participants

Policy information about studies involving human research participants and Sex and Gender in Research.

Reporting on sex and gender	A total of 4 non-covid19 infected patients, two males and two females, and 4 patients dead from covid19, two males and two females took part in this study. The detail of the chemotherapy was described in supplementary information files.
Population characteristics	A total of 4 non-covid19 infected patients, two males (BMI 34.5 kg/m2 aged 69, BMI 35.5 kg/m2 aged 74), and two females (BMI 26.9 kg/m2 aged 75, BMI 24.9 kg/m2 aged 61), and 4 patients dead from covid19, two males (BMI 27 kg/m2 aged 61, BMI 24.7 kg/m2 aged 55), and two females (BMI 26.9 kg/m2 aged 81, BMI 33.1 kg/m2 aged 57), took part in this study. The detail of the chemotherapy was described in supplementary information files.
Recruitment	For human studies, all autopsies were conducted at the risk-autopsy facility of the Department of Clinical Pathology/Cytology, Karolinska University Hospital, Huddinge, Stockholm, Sweden. All subjects were referred to the pathology department for the clinical autopsy to establish the precise cause of death. Some potential self-selection biases, such as sex, age, and BMI, in selecting the participants were not observed.
Ethics oversight	The study was approved by the Swedish Ethical Review Authority under the approval number DNR 2020-02446 and 2020-04339.

Note that full information on the approval of the study protocol must also be provided in the manuscript.

Field-specific reporting

Please select the one below that is the best fit for your research. If you are not sure, read the appropriate sections before making your selection.

[x] Life sciences   [ ] Behavioural & social sciences   [ ] Ecological, evolutionary & environmental sciences

For a reference copy of the document with all sections, see nature.com/documents/nr-reporting-summary-flat.pdf

Life sciences study design

All studies must disclose on these points even when the disclosure is negative.

Sample size	Sample numbers in vivo experiments were indicated according to our published data and other publications[1-3]. Due to the 3R principle for animal ethical permission, we used an optimal number of animals[4]. Sample sizes are indicated in Figure legends. No statistical calculations were used to predetermine sample size for in vivo experiments.<br><br>[1] Seki, T., et al. "Brown-fat-mediated tumour suppression by cold-altered global metabolism." Nature vol. 608,7922 (2022): 421-428.<br>[2] Seki, T., et al. "Ablation of endothelial VEGFR1 improves metabolic dysfunction by inducing adipose tissue browning." The Journal of experimental medicine vol. 215,2 (2018): 611-626.<br>[3] Iwamoto H., et al. Cancer lipid metabolism confers antiangiogenic drug resistance. Cell Metab. 2018 Jul 3;28(1):104-117.e5.<br>[4] Swedish 3R-center, https://jordbruksverket.se/languages/english/the-swedish-3rs-center
Data exclusions	No data were excluded.
Replication	All the biological experiments were performed at least twice and were reproducible. Murine studies: Experiments were performed at least twice. The infected experiments for different treatments were performed at least twice and were reproducible. Analysis for metabolites was performed once using five independent biological replecants. Human autopsy studies: the IHC and qPCR analyses using patients with Covid19 and non-Covid19 were performed two times using four independent biological samples.
Randomization	Age, gender, and background-matched mice and hamster were randomly allocated into the groups for all animal experiments.
Blinding	The author who was blinded to experimental groups performed the body weight of animal. Other experiments were carried out in non-blinding. Because the animals were kept in P3 Lab and accepted different treatment, it was not possible to hide the animal identity.

Reporting for specific materials, systems and methods

We require information from authors about some types of materials, experimental systems and methods used in many studies. Here, indicate whether each material, system or method listed is relevant to your study. If you are not sure if a list item applies to your research, read the appropriate section before selecting a response.

Materials & experimental systems

n/a	Involved in the study
☐	☒ Antibodies
☒	☐ Eukaryotic cell lines
☒	☐ Palaeontology and archaeology
☐	☒ Animals and other organisms
☒	☐ Clinical data
☒	☐ Dual use research of concern

Methods

n/a	Involved in the study
☒	☐ ChIP-seq
☒	☐ Flow cytometry
☒	☐ MRI-based neuroimaging

Antibodies

Antibodies used

Information for antibodies in this study as below,
- Rat anti-mouse CD31 antibody (MEC 13.3; 1:300; 553370, BD Pharmingen)
- Rabbit anti-mouse/human UCP1 polyclonal antibody (1:300; PA1-24894, Thermo Fisher Scientific) for IHC
- Rabbit anti-mouse/human COX4 polyclonal antibody (1:300; NB110-39115, Novus Biologicals) for IHC
- Goat anti-mouse Perilipin polyclonal antibody (1:300; NB100-60554, Novus biologicals) for IHC
- Rabbit anti-mouse polyclonal CA9 (1:300; NB100-417; Novus biologicals) for IHC
- Rabbit anti-mouse HIF1α antibody (D1S7W; 1:300; 36169, Cell Signaling Technology) for IHC
- Rabbit anti-mouse Ki67 polyclonal antibody (1:300; PA5-19462; Thermo Fisher Scientific)
- Alexa Fluor 555-labeled goat anti-rat antibody (1:300; A21434; Thermo Fisher Scientific)
- Alexa Fluor 488-labeled donkey anti-rat antibody (1:300; A21208, Thermo Fisher Scientific)
- Alexa Fluor 555-labeled donkey anti-rabbit antibody (1:300; A31572; Thermo Fisher Scientific)
- Alexa Fluro 488-labeled donkey anti-goat antibody (1:300; A11055; Thermo Fisher Scientific)
- Rabbit anti-mouse UCP1 polyclonal antibody (1:1000; PA1-24894, Thermo Fisher Scientific) for immunoblotting
- Rabbit anti-mouse COX4 polyclonal antibody (1:1000; NB110-39115, Novus Biologicals) for immunoblotting
- Rabbit anti-mouse polyclonal CA9 (1:1000; NB100-417; Novus biologicals) for immunoblotting
- Rabbit anti-mouse HIF1α antibody (D1S7W; 1:1000; 36169, Cell Signaling Technology) for immunoblotting
- Mouse anti-mouse beta-actin antibody (8H10D10; 1:2000; 3700; Cell Signaling Technology)
- Donkey anti-mouse IRDye 680RD antibody (1:15000, 926-68072, LI-COR Biosciences)
- Donkey anti-Rabbit IRDye 800CW antibody (1:15000, 926-32213, LI-COR Biosciences)

Validation

All antibodies used in this study were validated for the application and species by their manufacturers. The links as below,
- Rat anti-mouse CD31 antibody (MEC 13.3; 1:300; 553370, BD Pharmingen)
https://www.bdbiosciences.com/en-us/products/reagents/flow-cytometry-reagents/research-reagents/single-color-antibodies-ruo/purified-rat-anti-mouse-cd31.553370
- Rabbit anti-mouse/human UCP1 polyclonal antibody (1:300; PA1-24894, Thermo Fisher Scientific) for IHC
https://www.thermofisher.com/antibody/product/UCP1-Antibody-Polyclonal/PA1-24894
- Rabbit anti-mouse/human COX4 polyclonal antibody (1:300; NB110-39115, Novus Biologicals) for IHC
https://www.novusbio.com/products/cox4-antibody_nb110-39115
- Goat anti-mouse Perilipin polyclonal antibody (1:300; NB100-60554, Novus biologicals) for IHC
https://www.novusbio.com/products/perilipin-antibody_nb100-60554
- Rabbit anti-mouse polyclonal CA9 (1:300; NB100-417; Novus biologicals) for IHC
https://www.novusbio.com/products/carbonic-anhydrase-ix-ca9-antibody_nb100-417b
- Rabbit anti-mouse HIF1α antibody (D1S7W; 1:300; 36169, Cell Signaling Technology) for IHC
https://www.cellsignal.com/products/primary-antibodies/hif-1a-d1s7w-xp-rabbit-mab/36169
- Rabbit anti-mouse Ki67 polyclonal antibody (1:300; PA5-19462; Thermo Fisher Scientific)
https://www.thermofisher.com/antibody/product/Ki-67-Antibody-Polyclonal/PA5-19462
- Alexa Fluor 555-labeled goat anti-rat antibody (1:300; A21434; Thermo Fisher Scientific)
https://www.thermofisher.com/antibody/product/Goat-anti-Rat-IgG-H-L-Cross-Adsorbed-Secondary-Antibody-Polyclonal/A-21434
- Alexa Fluor 488-labeled donkey anti-rat antibody (1:300; A21208, Thermo Fisher Scientific)
https://www.thermofisher.com/antibody/product/Donkey-anti-Rat-IgG-H-L-Highly-Cross-Adsorbed-Secondary-Antibody-Polyclonal/A-21208
- Alexa Fluor 555-labeled donkey anti-rabbit antibody (1:300; A31572; Thermo Fisher Scientific)
https://www.thermofisher.com/antibody/product/Donkey-anti-Rabbit-IgG-H-L-Highly-Cross-Adsorbed-Secondary-Antibody-Polyclonal/A-31572
- Alexa Fluro 488-labeled donkey anti-goat antibody (1:300; A11055; Thermo Fisher Scientific)
https://www.thermofisher.com/antibody/product/Donkey-anti-Goat-IgG-H-L-Cross-Adsorbed-Secondary-Antibody-Polyclonal/A-11055

- Rabbit anti-mouse UCP1 polyclonal antibody (1:1000; PA1-24894, Thermo Fisher Scientific) for immunoblotting
https://www.thermofisher.com/antibody/product/UCP1-Antibody-Polyclonal/PA1-24894
- Rabbit anti-mouse COX4 polyclonal antibody (1:1000; NB110-39115, Novus Biologicals) for immunoblotting
https://www.novusbio.com/products/cox4-antibody_nb110-39115
- Rabbit anti-mouse polyclonal CA9 (1:1000; NB100-417; Novus biologicals) for immunoblotting
https://www.novusbio.com/products/carbonic-anhydrase-ix-ca9-antibody_nb100-417b
- Rabbit anti-mouse HIF1α antibody (D1S7W; 1:1000; 36169, Cell Signaling Technology) for immunoblotting
https://www.cellsignal.com/products/primary-antibodies/hif-1a-d1s7w-xp-rabbit-mab/36169
- Mouse anti-mouse beta-actin antibody (8H10D10; 1:2000; 3700; Cell Signaling Technology)
https://www.cellsignal.com/products/primary-antibodies/b-actin-8h10d10-mouse-mab/3700
- Donkey anti-mouse IRDye 680RD antibody (1:15000, 926-68072, LI-COR Biosciences)
https://www.licor.com/bio/reagents/irdye-680rd-donkey-anti-mouse-igg-secondary-antibody
- Donkey anti-Rabbit IRDye 800CW antibody (1:15000, 926-32213, LI-COR Biosciences)
https://www.licor.com/bio/reagents/irdye-800cw-donkey-anti-rabbit-igg-secondary-antibody

Animals and other research organisms

Policy information about studies involving animals; ARRIVE guidelines recommended for reporting animal research, and Sex and Gender in Research

Laboratory animals	Heterozygous K18-hACE c57BL/6J mice (strain: 2B6.Cg-Tg(K18-ACE2) 2Prlmn/J) at age of 12-week-old were purchased from the Jackson´s Laboratory (Strain #034860). Syrian hamsters at age of 8-week-old were purchased from JANVIER LABS.
Wild animals	This project did not use wild animals.
Reporting on sex	This project did not report on sex.
Field-collected samples	This study did not collect samples from the field.
Ethics oversight	All mouse studies were approved by the North Stockholm Animal Ethical Committee, Stockholm, Sweden

Note that full information on the approval of the study protocol must also be provided in the manuscript.
